# Habituation to visual onsets is affected by local and global distractors rate

**DOI:** 10.3758/s13414-023-02698-1

**Published:** 2023-03-28

**Authors:** Matteo De Tommaso, Massimo Turatto

**Affiliations:** 1 Center for Mind/Brain Sciences, University of Trento, Trento, Italy; 2 Department of Psychology, University of Milano-Bicocca, Milan, Italy

**Keywords:** Habituation, Statistical learning, Distractor location, Local inhibition, Visual onsets

## Abstract

Recent findings demonstrate that habituation of capture is stronger where onset distractors are frequent and weaker where they are rare, thus showing that habituation to onsets has a spatial selective nature. However, a debated question is whether habituation at a specific location is exclusively determined by the distractors’ local rate, or whether instead local habituation is also affected by the global rate of the distractors, which may occur also at other locations. Here, we report the results from a between-participants experiment involving three groups of participants exposed to visual onsets during a visual search task. In two groups, onsets appeared at a single location with a high 60% rate or a low 15% rate, respectively, whereas in a third group, distractors could appear in four distinct locations with the same 15% local rate, leading to a 60% global rate. Our results confirmed that locally, habituation of capture was stronger the higher the distractors rate. However, the key finding was that we found a clear and robust modulation of the global distractors rate on the local habituation level. Taken together, our results unambiguously show that habituation has both a spatially selective and a spatially nonselective nature.

## Introduction

The human cognitive system can learn environmental regularities and exploit this statistical information to achieve a more efficient analysis of the sensory input (Frost et al., 2019; Schapiro & Turk-Browne, 2015), including the ability to better ignore visual distractors where they are more likely to occur (Ferrante et al., 2018; Goschy et al., 2014; Wang & Theeuwes, 2018; Zhang et al., 2019).

Learning to ignore irrelevant stimuli that are repetitively encountered is a capacity that we share with different animal species, and that is described by the behavioral phenomenon of habituation (Harris, 1943; Thompson, 2009). Accordingly, previous studied have shown that human observers can habituate to salient visual stimuli recurrently appearing in their visual field, either as sudden onsets or color singletons (e.g., De Tommaso & Turatto, 2019; Turatto & Pascucci, 2016). Furthermore, and in line with one of its main features, habituation to visual onsets has been shown to be stronger the higher the distractors rate across different locations (Turatto & Pascucci, 2016), or when the distractors appear with different rates at different locations (Valsecchi & Turatto, 2022), thus mimicking what has been reported for color singleton distractors (Allenmark et al., 2019; Goschy et al., 2014; Kerzel et al., 2022; Lin et al., 2021; Sauter et al., 2018; Wang & Theeuwes, 2018). Crucially, the different capture habituation observed as a function of the distractors rate cannot simply be attributable to intertrial priming (Bogaerts et al., 2022), to the distractors temporal frequency or to the mere number of distractors being presented at different locations (Turatto & Valsecchi, 2023). Rather, in agreement with Sokolov’s model (Sokolov, 1960, 1963), habituation is controlled by the degree of the distractor expectation (Turatto & Valsecchi, 2023), so that the higher the rate of stimulation the less surprising the distractor becomes, leading to a stronger suppression of the orienting response. By contrast, at lower rates the distractor remains a relatively unexpected event, which continues to trigger a robust capture that is only partially attenuated.

The fact that habituation of capture is stronger at the locations or regions where the distractors appear more often (Allenmark et al., 2022; Turatto & Valsecchi, 2023; Valsecchi & Turatto, 2021)—namely, where they are more expected to occur, shows that habituation has a clear spatially specific component. However, when the distractors appear with different rates at two (or more) locations/spatial regions, the distractors rate has also a global component, which is the sum of the specific location rates. So, an interesting question is whether the global distractor rate can affect the local degree of habituation, or alternatively whether habituation is controlled only by the specific location rate. A first answer to this question has been provided by Valsecchi and Turatto (2021), who presented participants with a color singleton distractor appearing with three different rates, high, medium and low, at three separated locations. After the end of the training phase, when habituation was stronger at the locations with the higher distractor rates, the distractor rates at all locations were equalized at the lower rate. This resulted in a recovery of capture at the locations where the distractor had appeared at the higher level, which is consistent with the fact that at those locations the distractor rate was substantially reduced compared with the training phase. Crucially, however, a recovery of capture was also observed at the lower rate location, where the distractor rate remained unaltered. Valsecchi and Turatto (2021), proposed that the latter unexpected finding was explained by the fact that the lowering of the distractor rate at the previous high and medium rate locations reduced the global distractor rate, which in fact modulated the degree of habituation of capture at the local level.

More recently, on the basis of a series of experiments Allenmark et al. (2022) reached a different conclusion, namely that habituation of feature-singleton distractor is only location specific. The authors reported that the interference of distractors appearing with the same rate in a specific region is substantially equivalent across groups designated with a variety of global distractor conditions. A similar finding was reported by Turatto and Valsecchi (2023) with onset distractors, as they found that the degree of habituation of capture obtained with an onset appearing in a fixed position with a 33% rate was basically similar, although slightly weaker, to that observed when the same onset appeared at two distinct locations, each with a 33% rate, which summed up to a 66% global rate.

In light of this apparently contradictory scenario we decided to design a straightforward between-subjects experiment to directly test whether habituation is only dictated by the local rate of distractors occurrence, or alternatively whether habituation is also affected by the distractors global rate. Three groups of participants underwent the same visual discrimination task, while trying to ignore an onset distractor that appeared in the periphery (also see Turatto & Valsecchi, 2023). For the *local low-rate* (LL) group, the distractor appeared at a fixed location with a 15% rate; for the *local high-rate* (LH) group, the distractor also appeared at a fixed location but with a 60% rate; finally, for the *global high-rate* (GH) group the distractor appeared with a 15% rate at each of four positions, for a 60% global rate. Under these conditions, we expected a stronger habituation in the LH group compared with the LL group, a result that would replicate previous studies (Müller et al., 2009; Turatto & Pascucci, 2016; Turatto & Valsecchi, 2023; Won et al., 2019). However, the key comparisons will regard the degree of habituation in the LL group versus the GH group, and in the LH group versus the GH group. If habituation is strictly location specific (Allenmark et al., 2022), no difference should emerge between the LL and the GH groups, as in both groups the local distractor rate is the same (15%); by contrast, if habituation is also affected by the global distractor rate (Valsecchi & Turatto, 2021), then habituation in the GH group should be larger than in the LL group, despite the same local rate (15%), and possibly weaker than in the LH group because locally the distractor rate is lower in the global condition (15% in the GH vs. 60% in the LH).

## Methods

### Sample-size justification

Based on the design of the present study, evidence for a global capture habituation can be obtained from a difference between the amount of attention capture generated by the distractor across three groups with different distractor rates. We estimated the effect size to be expected based on the study from Turatto and Valsecchi (2023), which reports the results from an online experiment (Exp. 1) in which two groups of participants were exposed to onset distractors occurring at different rates in a task very similar to the one adopted in the present experiment, and showing that the effect size of the attentional capture across rates (66% vs. 33%; Turatto & Valsecchi, 2023) was ${\eta}_p^2$ = .193. Because in the present study the difference between the higher and lower distractor rates (60% vs. 15%) is larger, we expect the effect to be bigger. However, here we evaluated three conditions, therefore we decided to adopt a more conservative perspective by estimating a smaller effect size (${\eta}_p^2$ = .15). Therefore, an a-priori power analysis indicated a total sample size of *N* = 60 with *α* = .05 to achieve a power of 80% (G*Power; Faul et al., 2007).

### Participants

Participants (*N* = 60, *M*_*age*_ = 27.1 years, 30 females) were recruited online via Prolific (Prolific Academic Ltd, Oxford, UK). In order for instructions to be fully comprehended, being fluent in the English language and having no literacy difficulties were required for participation. Further criteria were to have normal or corrected-to-normal vision, and to be naïve with regard to the experiment (individuals who participated in previous similar studies were not recruited). Two participants were excluded from the analyses due to low overall accuracy (<75%).

### Apparatus

The experiment was programmed in PsychoPy Software (Peirce, 2007) and run online via the Pavlovia Platform (Open Science Tools Limited, Nottingham, UK). No tablets or smartphones were allowed to perform the task, which was run on a personal computer.

### Stimuli and procedure

Stimuli dimensions are reported as degrees of visual angle assuming a monitor height of 34 cm and a viewing distance of 60 cm. However, since participant potentially performed the task with monitors of different size, the dimensions of the stimuli were scaled according to the monitor’s sizes and the experiment was run in full-screen mode. Scaling was implemented automatically by the software through a specific unit of measurement, which scaled the size of the stimuli relative to the participants’ screen (i.e. “height units”).

**Fig. 1:**
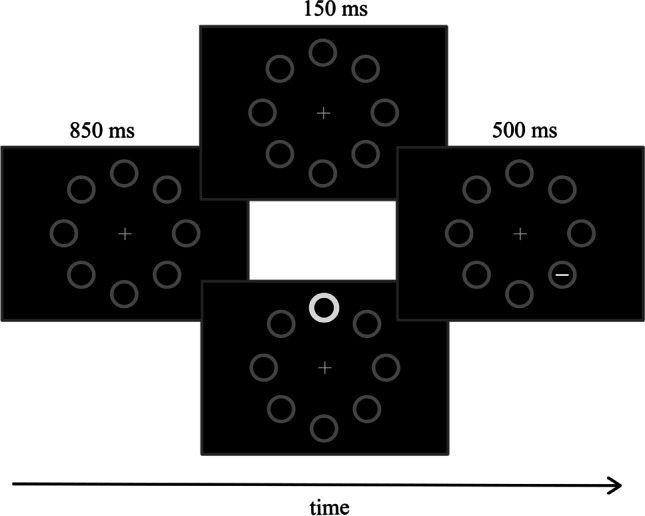
Schematic representation of the trial events in the experiment (see Methods for details). Participants’ task was to discriminate the orientation (vertical vs. horizontal) of the target line. In distractor-present trials, the distractor appeared 150 ms before the target. (See the Methods section for details about the distractor rate in the different groups of participants.)

Every trial began with a white fixation cross (0.3° × 0.3°) appearing over a black background and positioned at the center of the screen. After 1,000 ms, eight equidistant gray circles (3° diameter, 0.1° thick) appeared at 10° of eccentricity from the fixation cross. After that, 1,000 ms were elapsed, the target line (0.75° length, 0.1° thick) appeared inside one of four circles positioned along the oblique meridians. In distractor-present trials, 150 ms before the target appearance, one of the four circles positioned along the vertical or horizontal meridians became abruptly white and increased its thickness to 0.3° (see Fig. 1). Participants’ task was to report as quickly and as accurately as possible the orientation of the target line by pressing the down arrow of the keyboard if the line was vertical, or the right arrow if it was horizontal. Participants were given 1,500 ms for responding from the target onset, which remained on the screen for a maximum of 500 ms or until a response was emitted. If the response was incorrect or exceeded the time limit, a red message appeared on the screen (“Error” or “Try to be faster!”) for 800 ms. The intertrial interval set to 500 ms, during which the screen was black. The task consisted of 480 trials equally divided in four blocks, with an additional distractor-absent practice session of 20 trials. Distractor presence was randomized across trials according to the following conditions.

Participants were equally distributed across three groups which defined the respective experimental conditions. As anticipated before, in the local low-rate group (*N* = 20), and in the local high-rate group (*N* = 20), the distractor appeared in 15% and 60% of the total trials, respectively, always in the same location, which was constant throughout the task and balanced across participants. In the global high-rate group (*N* = 20), the distractor appeared in each of the possible four positions, with a local rate of 15%.

All participants received detailed instructions concerning the task and were informed about the general aim of the experiment through the Prolific interface. They gave their consent by agreeing to be redirected to the experiment URL. Participants were paid 8 £/h for their participation, and the experiment lasted approximately 30 minutes. The experiment was carried out in accordance with the Declaration of Helsinki.

## Results

The analyses were performed with custom-written scripts in MATLAB and JASP (Version 0.16.4). Response times (RTs) outliers (2.2%) for correct trials (overall accuracy 94.8%) were identified and excluded using the procedure suggested by Cousineau and Chartier (2010). For null-hypothesis significance testing, Greenhouse–Geisser correction was applied to the degrees of freedom when sphericity assumption was violated. Post hoc *t* tests were Bonferroni corrected for multiple comparisons. Bayes factors were estimated quantifying how much more likely the data were under the alternative hypothesis than under the null hypothesis (*BF*_*10*_). For more than one predictor we estimated the inclusion Bayes factor across matched models (*BF*_*incl*_; van den Bergh et al., 2020). Posterior odds were corrected for multiple comparisons.

**Fig. 2:**
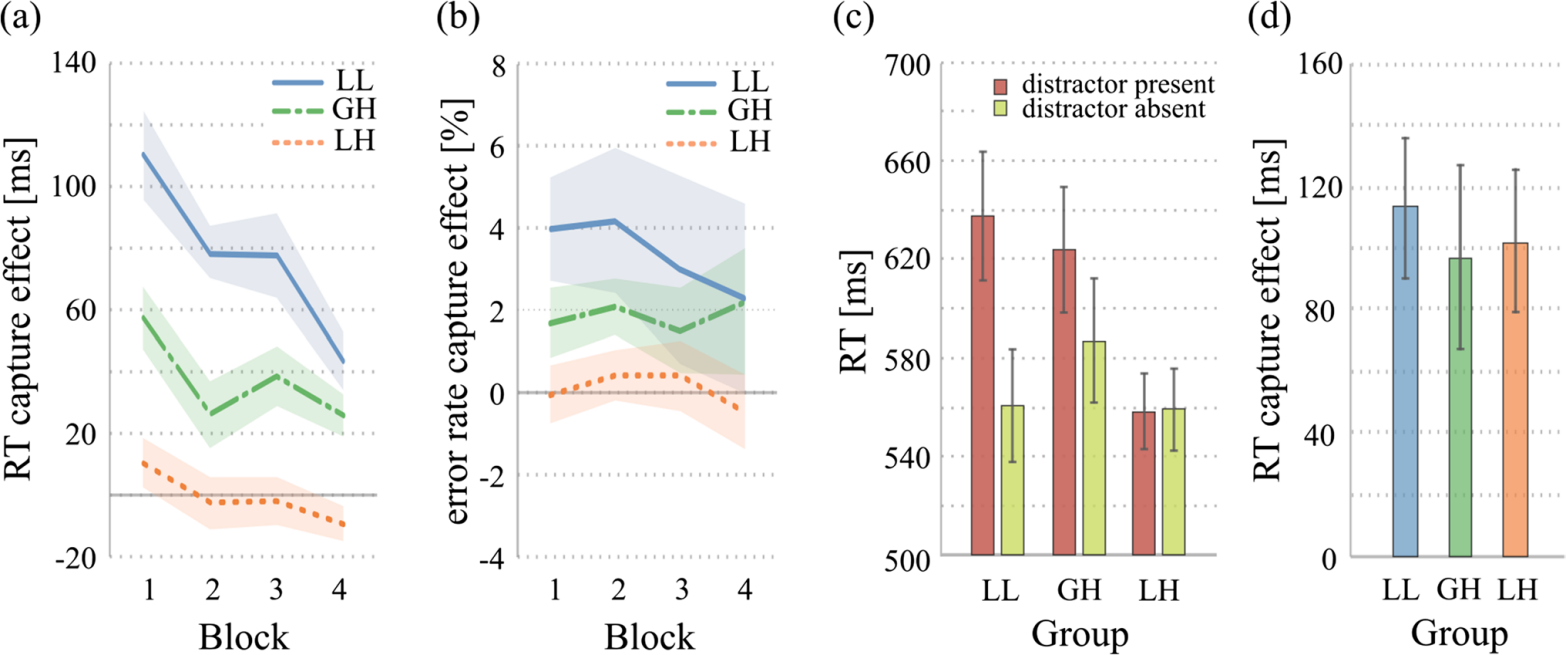
Results of the experiment (LL = local low rate; GH = global high-rate; LH = local high rate). **a** RT capture effect as a function of Block and Group. **b** Error rate capture effect as a function of Block and Group. **c** Absolute RTs as a function of group and distractor presence. **d** RT capture effect at the beginning of training (corresponding to the first six distractor presentations) as a function of Group. Error bars represent *SEM*. (See Methods for details.) (Color figure online)

We calculated for each participant the attentional capture effect defined as a positive RT difference when RTs in the distractor-absent trials are subtracted from RTs in the distractor-present trials. The resulting data were entered in a repeated-measures analysis of variance (ANOVA), with Block (1 to 4) and Group (LL, LH, GH), which showed a main effect of Block, *F*(2.69, 147.9) = 16.5, *p* < .001, ${\eta}_p^2$ = .231, *BF*_*incl*_ = 1.8×10^6^, a main effect of Group, *F*(2, 55) = 25.8, *p* < .001, ${\eta}_p^2$ = .484, *BF*_*incl*_ = 9.9×10^5^, and a significant interaction, *F*(5.38, 147.9) = 2.40, *p* = .036, ${\eta}_p^2$ = .080, *BF*_*incl*_ = 1.25 (see Fig. 2a). Post hoc comparisons confirmed that the three conditions differed significantly between each other: LL vs. LH, *t*(37) = 7.18, *p* < .001, *d* = 1.81, *BF*_*10*_ = 2.85×10^16^; LL vs. GH, *t*(36) = 3.64, *p* = .002, *d* = 0.931, *BF*_*10*_ = 4.28×10^3^; GH vs. LH, *t*(37) = 3.49, *p* = .003, *d* = 0.882, *BF*_*10*_ = 9.07×10^5^.

The same analysis on error rates resulted in a significant main effect of condition, *F*(2, 55) = 5.33, *p* = .008, ${\eta}_p^2$ = .162, *BF*_*incl*_ = 2.38, but no other significant effects (Block, *p* = .834, *BF*_*incl*_ = 0.030; Block × Group, *p* = .970, *BF*_*incl*_ = 0.021; see Fig. 2b). This analysis confirmed that the pattern emerged from RTs was not due to a speed–accuracy trade-off, and that the amount of capture habituation was weaker in the LL condition, intermediate in the GH condition, and stronger in the LH condition.

Since the distractor-absent RTs could be potentially influenced by the distractor frequency (e.g., Müller et al., 2009), we entered these RTs in a repeated measures ANOVA with Block (1 to 4) and Group (LL, LH, GH). The results showed a main effect of Block, *F*(2.40, 132.4) = 18.7, *p* < .001, ${\eta}_p^2$ = .254, *BF*_*incl*_ = 7.40×10^7^, but no other significant effect (Group, *p* = .613, *BF*_*incl*_ = 0.493; Block × Group, *p* = .697, *BF*_*incl*_ = 0.049; see Fig. 2c). The same analyses on distractor-absent error rates yielded the same effects: Block, *F*(2.59, 142.9) = 6.95, *p* < .001, ${\eta}_p^2$ = .112, *BF*_*incl*_ = 1.36×10^2^; Group, *p* = .906, *BF*_*incl*_ = 0.195; Block × Group, *p* = .934, *BF*_*incl*_ = 0.023.

Given that the difference between the three habituation conditions was crucial to support the conclusion that the global distractors rate modulate the degree of habituation at the specific location, we wanted to collect further evidence showing that the three groups did not differ in the first place in their habituation capacity. In other words, the three groups should show the same degree of capture at the beginning of training, which then was subject to a different degree of habituation as a function of the distractors rate. To this aim, we confined our analysis of the capture effect at the beginning of training, and the three groups were matched in terms of number of distractors seen. We thus considered the first six distractor-present trials for each group, and the corresponding mean RTs was confronted with the mean RTs from the first four distractor-absent trials in the LH and GH groups (60% rate), and with the mean RTs from the first 34 distractor-absent trials in the LL group (15% rate). The resulting initial capture values were entered into a one-way ANOVA, with Group as the only factor, which was not significant (*p* = .891, *BF*_*incl*_ = 0.152), thus showing that the amount of capture was equivalent in the three groups at the beginning of training (see Fig. 2d).

## General discussion

The present findings were clear cut, showing that habituation to onset distractors, and also to feature-singleton distractors, is dictated both by the local and the global distractors rate.

This conclusion arises from the following observations. For the same local distractor probability (15%), habituation of capture is stronger (i.e., capture is weaker) when the global distractor rate is higher (LL vs. GH group). This result may have two possible explanations: Either the effect of the global distractor rate (60%) adds to the effect of the local distractor rate (15%), though not necessarily in a linear fashion, or more parsimoniously the degree of habituation is determined only by the global distractor rate, which was higher in the GH group compared with the LL group. The latter scenario seems unlikely, given that Turatto and Valsecchi (2023) have already shown that habituation to onset varies as a function of the local distractor rate. What clearly emerges from the LL vs. GH comparison, instead, is that habituation is not driven solely by the local distractor rate when the distractor appears at different locations (also see Valsecchi & Turatto, 2021). Interestingly, we also found that for the same global distractor rate (60%) habituation was stronger when the distractor appeared at a fixed location than at four locations (LH vs. GH group). This clearly shows that habituation is not simply determined by the global distractor rate, because otherwise the LH and GH groups should have been equivalent. By contrast, the fact that habituation in the LH group was significantly stronger than in the GH group again suggests that the local and global components operate in tandem in determining the overall level of habituation. Indeed, whereas in terms of the global rate (60%) the two groups were equivalent, at the local level the distractor rate was higher in the LH group (60%) than in the GH group (15%).

While the location-specific habituation is determined by the local distractor rate, the global distractor rate could affect habituation in two possible ways: one possibility is that the single local rates are somehow integrated, thus increasing the level of habituation compared with that achievable at a single location; the other possibility is that the global effect, which is added on top of the local effect, arises from a habituation occurring at a purely feature-based level, where its degree is simply proportional to the overall distractor rate. This feature-based habituation might correspond to a uniform suppression applied across all locations in topographically organized feature maps (also see Zhang et al., 2019), or in nontopographic feature representations, at a higher level than the saliency/priority map (Koch & Ullman, 1985).

At any rate, the present findings are fully in agreement with the study of Valsecchi and Turatto (2021) with color singleton distractors, and are also compatible with the work of Turatto and Valsecchi (2023) with onset distractors, where a trend toward a possible effect of the global distractor rate on the local degree of habituation was evident. What we found here appears instead in contrast with the study of Allenmark et al. (2022) with orientation singleton distractors, who concluded that habituation is controlled only by the local distractor rate. The discrepancy with the study of Allenmark et al. (2022) might however be more apparent than real. Indeed, in spite of the authors strong claim that habituation is only location specific, as a matter of fact their results revealed that, at least numerically, a small global habituation effect was evident. Moreover, the results also showed that the distractors habituation within the specific lower-rate region was not homogenous, since at the region borders it was affected by the distractor probability in the adjacent higher-rate region, thus suggesting that habituation was not solely driven by the distractors rate at the specific location.

Neuroimaging data also seem to support a scenario compatible with our proposal, according to which the degree of habituation is affected by both the local and the global distractor rate. So, although local habituation is associated with a down-modulation in spatially specific receptive fields in the early visual cortex (Zhang et al., 2022), signs of distractor rejection in the same cortex are found also when the distractor appearance is not tied to a specific spatial location, but the distractors occur unpredictably at various (global) locations (Won et al., 2020). This result favors the hypothesis that lower-level suppression may originate from, or at least is influenced by, a more global analysis of the distractor spatial distribution, which can only take place at higher-level visual areas, where neurons have more broader receptive fields, necessary to evaluate the global distractors rate (Adam & Serences, 2021).

However, regardless of the interpretations of the existing neuroimaging evidence, the behavioral results that we have reported straightforwardly and unambiguously show that habituation is controlled both by the local and the global distractor rate (Turatto & Valsecchi, 2023; Valsecchi & Turatto, 2021), which does not imply that their contribution is symmetrical.

## Data Availability

All data and codes for analyses have been made publicly available via OSF and can be accessed online (https://osf.io/j25fy/?view_only=367dc2a87a6e4d2eb38813bdee6289af). The design and analysis plans for the experiment were not preregistered.
